# Non-invasive image-based method for mass estimation of caterpillars of Lepidoptera

**DOI:** 10.1093/jisesa/ieag088

**Published:** 2026-08-20

**Authors:** Robin von Allmen, A Blair Stokes, Arvid Hellesöy Annell, Christopher Wheat

**Affiliations:** Department of Zoology, Stockholm University, Stockholm, Sweden; Department of Biological Sciences, University of South Carolina, Columbia, South Carolina, USA; Department of Zoology, Stockholm University, Stockholm, Sweden; Department of Biological Sciences, University of South Carolina, Columbia, South Carolina, USA

**Keywords:** computer vision tool, automatic measurements, caterpillar physiology, continuous monitoring

## Abstract

Live biomass is a key metric for monitoring insect development and survival, especially of larval species of Lepidoptera. Nonetheless, manual weighing is labor-intensive, can induce stress, and may introduce experimental bias. Here, we present a non-invasive, open-source ImageJ macro that enables repeated measurements of caterpillar silhouette area using computer vision tools. We validated this approach using larvae of five different lepidopteran species and found a strong correlation between the silhouette area and live biomass. The macro achieved high detection accuracy (∼98%) and was approximately six times faster than manual weighing. This approach can potentially allow for continuous monitoring of larval biomass while reducing handling and associated stress, providing a practical tool for experimental studies of Lepidoptera.

## Introduction

Estimation of insect growth rate requires weight measurements over time, as is commonly used to study caterpillar feeding performance. Repeated weighing of individuals throughout their development is undesirable and potentially harmful, as frequent manual handling of caterpillars, especially during the first instar, can induce stress, disrupt normal growth and development, and ultimately compromise experimental outcomes (Smiley and Wisdom 1982, Oonincx et al. 2025). In addition, individually weighing large numbers of caterpillars or insect larvae generally imposes substantial labor (Sample et al. 1993), which makes growth monitoring at short-time intervals unfeasible.

Despite these constraints, larval biomass remains a key metric in developmental and ecological studies of Lepidoptera (Griese et al. 2020, Valsamakis et al. 2020). The biological relationship between the morphology and body mass of larvae is already well established. Rogers et al. (1977) describe that the larval length follows a robust power–law relationship with weight, which was later confirmed for a broad range of insect families (Sample et al. 1993). Subsequent work further demonstrated that incorporating body width, particularly its segment-specific variation along the larval body, further improved the predictive accuracy (Smiley and Wisdom 1982, Mocq et al. 2024, Sample et al. 1993) of such models. Together, these findings emphasize that accurate, non-invasive size measurements can provide reliable proxies for larval weight.

Despite this, a clear research gap persists. Previous studies have largely relied on geometric formulas to estimate larval volume from length and width (Smiley and Wisdom 1982, Gruner 2003, Majewski et al. 2024, Mocq et al. 2024), which can introduce additional measurement errors as multiple parameters need to be measured at once. Moreover, such approaches either focus on detailed and high-precision measurements of individual larvae (Smiley and Wisdom 1982, Sample et al. 1993, Gruner 2003) or large-scale setups designed for high-throughput assessments of body mass and survival within the agricultural and food industry, without feasibly identifying individuals through a continuous monitoring scheme (Majewski et al. 2024). To our knowledge, the potential of the silhouette area, derived from the number of pixels in an image, as a predictor of larval biomass has remained largely unexplored.

Here, we present an open-source tool that automates the estimation of caterpillar weights using computer-vision tools in FIJI (Data Availability Statement). The macro handles both multi Petri dish setups and individual measurements. Our approach minimizes handling, reduces labor and measurement bias, and enables high-frequency tracking of larval growth using simple equipment commonly found in research labs. We used five Lepidopteran species reared under controlled laboratory conditions to test the relationship between silhouette area and weight, and to validate the detection accuracy of the macro.

## Materials and Methods

### Lepidoptera Species

Five Lepidopteran species were reared to represent a broad spectrum of phenotypic and developmental variation. All ­species were reared on their respective host plants: *Colias croceus* (Geoffroy, 1785; Lepidoptera: Pieridae) on *Vicia villosa* (Fabaceae), *Pieris brassicae* (Linnaeus, 1758; Pieridae) on *Armoracia rusticana* (Brassicaceae), *Actius luna* (Linnaeus, 1758; Saturniidae) and *Hyalophora cecropia* (Linnaeus, 1758; Saturniidae) on *Liquidambar styraciflua* (Altingiaceae), and *Hemileuca sandra* (Pavulaan, 2020; Saturniidae) on *Quercus falcata* (Fagaceae). Larvae were fed ad libitum.

*C. croceus* and *P. brassicae* larvae were kept on a 20:4 light: dark cycle at 22 °C. *Actius luna*, *H. cecropia*, and *H. sandra* larvae were kept on a 16:8 light: dark cycle at 27 °C and 15 °C at night. Selected larvae represented individuals of various sizes and instars to ensure that the automated workflow could accurately account for individual developmental variation (Mocq et al. 2024). All individuals were only measured once.

### Silhouette Area Measurements

**Fig. 1.**
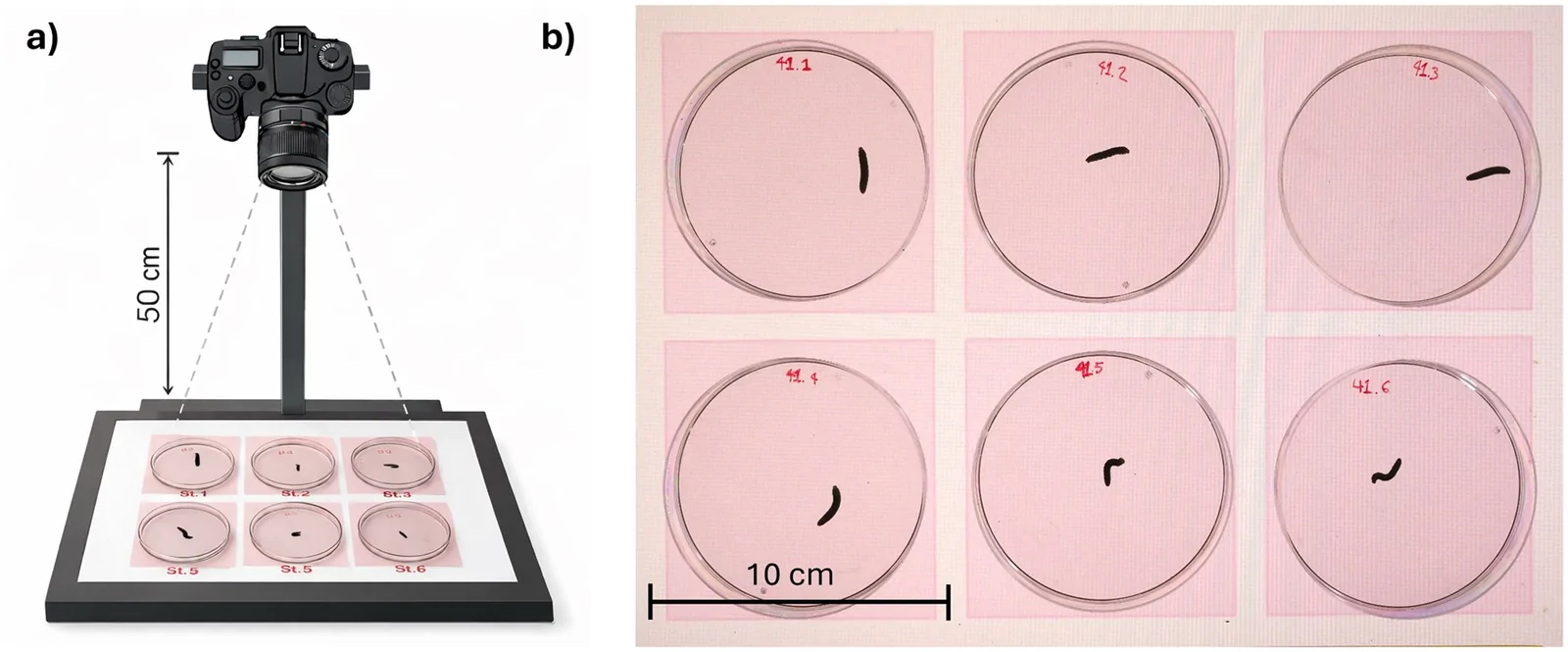
Experimental setup for imaging. A) Schematic representation of the imaging arrangement, showing six Petri dishes placed on an illuminated screen and photographed from above. B) Example image captured during imaging, showing the six Petri dishes used for measuring caterpillar silhouette area.

Images were captured on three separate occasions using either an Olympus OM-D camera with a 25 mm 1:1.2 lens or a Canon EOS 1500D camera with an EF-S 18–55 mm F 3.5–5.6 IS II lens positioned approximately 50 cm above the Petri dishes, yielding a resolution of ∼0.07 mm per pixel (Fig. 1A). To calibrate the image scale in millimeters per pixel, an initial reference image was taken without caterpillars using a ruler placed along the lower edge of the frame as a reference. Subsequently, up to 10 labeled Petri dishes, each containing a single caterpillar, were arranged on an illuminated, white background, with their positions indicated by colored rectangles (Fig. 1B).

Using an HSV threshold, each marked region in the original images (Fig. 1B) was segmented and processed individually. An edge detection-based algorithm then identified larger-bodied objects within the Petri dish region. The “Analyse Particles” function was used to measure the detected objects. A size and shape filter removed objects either smaller or larger than expected, as well as objects deviating from the rounded morphology of the caterpillar. For each Petri dish, the ranked position, calibrated scale and the estimated projected area (ie the area of the caterpillar’s silhouette in dorsal view) of each caterpillar were exported to an Excel file. Finally, the masks corresponding to each Petri dish region were saved in a separate folder for experimental validation.

### Statistical Analysis

The segmentation accuracy of the macro was evaluated using recall and precision. Recall was defined as the proportion of photographed caterpillars that were correctly detected by the algorithm, ie true positives divided by the sum of true positives and false negatives. Precision was defined as the proportion of detected objects that corresponded to caterpillars, ie true positives divided by the sum of true positives and false positives.

We further distinguished between coiled (ie tail and head are touching), small (ie silhouette area less than 10 mm^2^) and edge individuals (ie touching the rim of the Petri dish) to compare the odds-ratios of different groups not being detected. As the number of not detected individuals was small, we used a pairwise Fisher’s exact test to test for significant differences between groups.

The area of the caterpillar, in square millimeters, was calculated based on the number of pixels (n) and the per-pixel resolution in millimeters (equation 1):


(1) $$\mathrm{Area}\;[{mm}^{2}]=n\;\times {\mathrm{scale}}^{2}$$

The relationship between Area, in square millimeters, and weight in milligrams was described using a power model (Rogers et al. 1977), with the following form:


(2) $$y=\;a\times {\left(x\right)}^{b}$$

where  y equals live biomass, x is the calculated area in square-millimeters (equation 1) and a and b are regression coefficients. Both weight and area were transformed to natural logarithms for a regression analysis:


(3) ln (y) =ln (a) +b×ln⁡(x)

All statistical analyses were performed in R (version 4.5.1) using the stats (version 4.5.1), lmtest (version 0.9–40) and performance (version 0.15.2) packages. One data point was removed, which was confidently identified as an outlier due to a measuring error.

A linear regression model was fit to equation (3) using the natural log-transformed weight as the response variable and the natural log-transformed silhouette area together with the species as independent variables. To evaluate the predictive error of the linear regression model, we performed a 10-fold cross-validation. The data was divided into 10 folds, with the model fitted on 9 folds and evaluated on the remaining fold. This procedure was repeated until each fold had served once as the validation dataset, ensuring that each larva was predicted once by a model fitted without including that individual. Prediction accuracy was assessed by comparing measured and predicted weight values using the mean absolute percentage error (MAPE), and cross-validated *R*². Where the linear regression model was fitted on a transformed scale, predictions were back-transformed before calculating prediction errors on the original weight scale.

## Results

### Imaging and Detection Accuracy

To evaluate the detection accuracy of our workflow, we photographed a total of 740 Petri dishes of which 400 were of *C. croceus* and 340 contained *P. brassicae*, each containing one individual caterpillar. Per Petri dish, we spent approximately 1 min for manual weighing, while imaging and the automated workflow amounted to ten seconds per caterpillar. Of all photographed individuals, 732 were detected and measured automatically. For eight caterpillars, of which three were coiled, one was placed along the edge of the Petri dish, and four were small in size, the overview images needed to be manually cropped and processed individually. In addition, 34 non-caterpillar objects, including fecal matter, dust, and sample labels, were incorrectly detected as caterpillars. Overall, the FIJI macro, therefore, achieved an average recall and precision of 98.9% and 95.5%, respectively. Pairwise Fisher’s exact tests showed higher missed-detection probabilities for coiled, edge-positioned, and small caterpillars than for normal caterpillars, while the groups did not differ from each other (all *p*adj = 1.00).

### Area and Weight Relationship

A total of 231 caterpillars at different developmental stages and instars were weighed, with weights ranging from 1.7 to 3,971.2 milligrams of which 51 belonged to *A. luna*, 58 to *C. croceus*, 36 to *H. sandra*, 27 to *H. cecropia* and *P. brassicae*, respectively. These caterpillars were then photographed based on our automated workflow (see the section “Silhouette Area Measurements”).

**Fig. 2.**
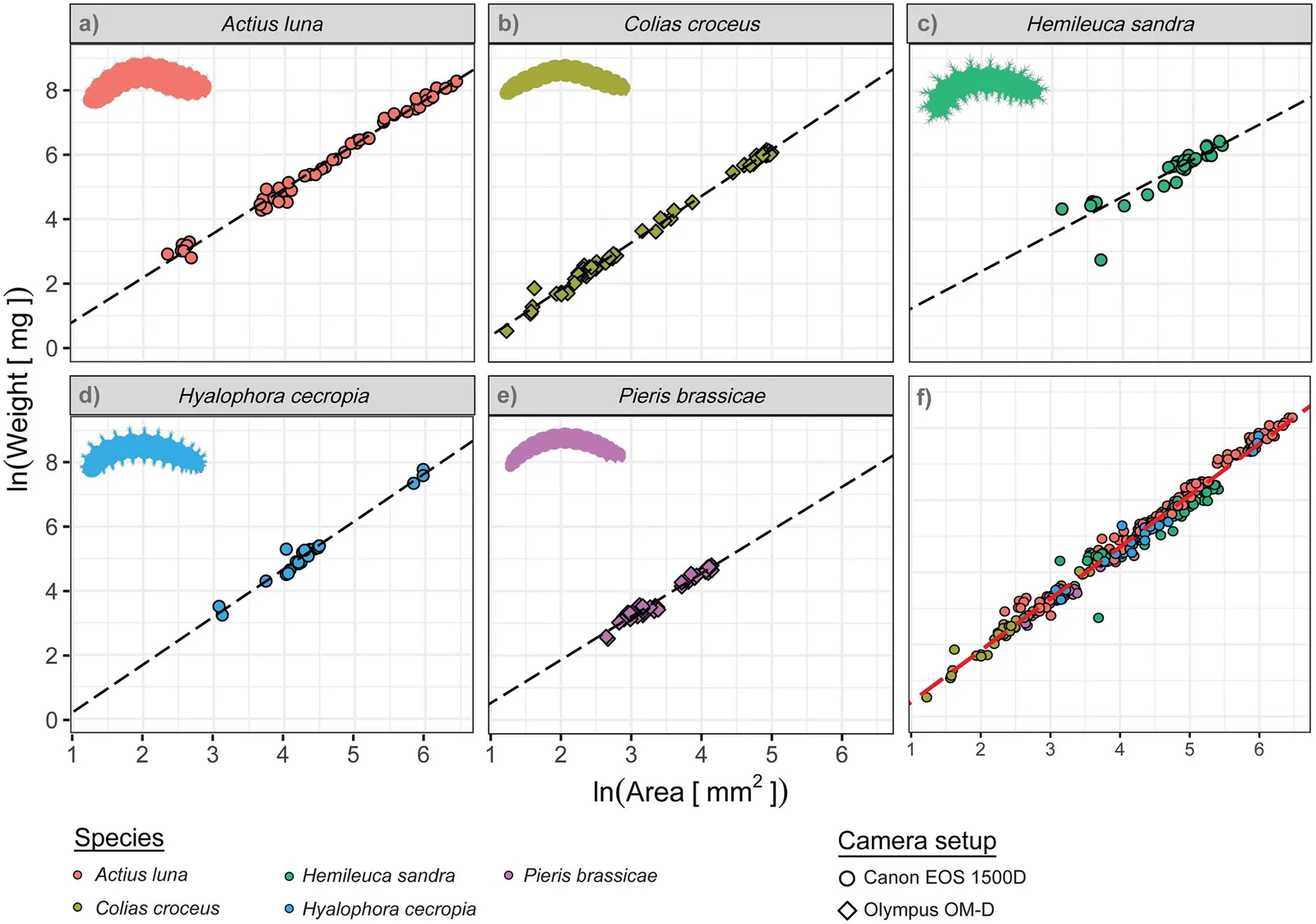
Relationship between caterpillar silhouette surface area and live biomass across five lepidopteran species. Points represent individual caterpillar measurements, with colors indicating species and symbols indicating the camera setup used for image acquisition. Both silhouette surface area and live biomass were natural log transformed. Panels show species-specific relationships for A) *Actias luna*, B) *Colias croceus*, C) *Hemileuca sandra*, D) *Hyalophora cecropia*, and E) *Pieris brassicae*. Panel F) shows the pooled relationship across all species. Dashed black lines indicate species-specific fitted linear regressions, while the solid red line in panel F) indicates the fitted regression across the full dataset.

A log-linear regression corresponding to a power–law model was fitted to assess the relationship between silhouette area and body mass across five species, with species identity and its interaction with silhouette area included as predictors. The model explained most of the variation in body mass (*R*² = 0.987, adjusted *R*² = 0.986) and revealed a highly significant relationship between silhouette area in square millimetres and body mass in milligrams (*F*_9,221_ = 1,824, *P* < 0e-15; Fig. 2). The fitted power–law relationships differed among species, with both the estimated intercepts and scaling exponents varying across taxa (Table 1). *H. sandra* exhibited the highest estimated intercept, whereas *H. cecropia* showed the lowest. The estimated scaling exponent also varied among species, with *H. cecropia* and *C. croceus* exhibiting steeper area–mass relationships than *A. luna*, while *H. sandra* showed a shallower relationship. In contrast, the estimated coefficients for *P brassicae* were more similar to the reference species. Overall, the model captured species-specific differences in the area–mass relationship while maintaining an excellent overall fit (Table 1). In addition, residual variation did not differ among species and caterpillar positions overall (*R*² = 0.049, adjusted *R*² = 0.023; *F*_6,223_ = 1.90, *P *= 0.082), with only *Colias croceus* showing significantly lower log-transformed absolute residuals than the reference species (β = −0.541, SE = 0.219, *t *= −2.47, *P *= 0.014).

**Table 1.** Species-specific coefficients of the power–law model ­(equation 2) derived from the log-linear regression model (equation 3) describing the relationship between silhouette area and body mass

Species	a	b	*P*-value
***Actius luna***	**0.556 ± 0.048**	**1.385 ± 0.018**	**<2 × 10^−16^**
***Colias croceus***	**0.344 ± 0.047**	**1.445 ± 0.031**	**0.01469**
***Hemileuca sandra***	**2.248 ± 0.557**	**1.009 ± 0.052**	**5.06 × 10^−13^**
***Hyalophora cecropia***	**0.275 ± 0.064**	**1.493 ± 0.051**	**0.02579**
***Pieris brassicae***	0.440 ± 0.084	1.342 ± 0.050	0.35224

Coefficient (a) represents the back-transformed intercept, while coefficient (b) represents the slope of the log-linear relationship. Bold values indicate coefficients that differ significantly from the reference species, *A. luna*. The *P*-value refers to the significance of the interaction term between species identity and silhouette area, testing whether the slope differs from that of *A. luna*.

Ten-fold cross-validation confirmed high predictive performance of the log-transformed area and weight relationship across all 231 larvae. The cross-validated *R*² on the log scale was 0.985, while the back-transformed predictions deviated from observed biomass by an average of 14.3%.

## Discussion

The FIJI macro performs with high accuracy and efficiency, with only a few individuals or Petri dishes being missed. We additionally experienced that increasing contrast of the colored rectangles, as well as constant imaging conditions, improve the detection accuracy of Petri dishes and caterpillars subsequently. Misidentifications more consistently happened for individuals that were small in size, touching the edge of the Petri dish, or coiling up. We, therefore, recommend moving caterpillars, if necessary, from the edge, adjusting the threshold parameters in the FIJI macro and uncoil caterpillars before capturing the image.

Our results show a strong relationship between silhouette area and body weight across the five studied species, with the log-linear power–law model explaining 98.7% of the variation in body mass. This is consistent with previous studies on lepidopteran larvae, which also reported tight size–biomass relationships (Smiley and Wisdom 1982, Gruner 2003, Majewski et al. 2024). The scaling factor (b) of our power–law model (Fig. 2F, a = 0.328, b = 1.457) closely matches published values of other lepidopteran larvae, for example, Gruner (2003: a = 0.061, b = 1.374, *R*^2^ = 0.877), Smiley and Wisdom (1982: a = 0.631, b = 1.03, *R*^2^ = 0.926) and Sample et al. (1993: a = 0.043, b = 1.483, *R*^2^ = 0.96), indicating that the underlying scaling relationship is highly consistent across studies. In contrast, our intercept (a) differs from earlier work, likely due to differences in how silhouette area was estimated. Further, the differences in absolute residual distances across species can be explained by interspecific differences in morphology, posture, size range (Lin et al. 2011), and image-segmentation quality, all of which may influence the accuracy with which silhouette area predicts body mass.

We expect that this open-source tool will facilitate laboratory workflows by enabling accurate weight estimation and supporting continuous, non-invasive monitoring of larval development and growth.

## Conclusion

Computer vision tools can significantly reduce laboratory workload, measurement error, and manual handling of test subjects. Further improvements in both image acquisition and computational tools, such as machine learning models, may in the near future further improve automation and allow for real-time monitoring of larval development. In addition, establishing the relationship between silhouette area and body weight for other insect larvae, especially in species with instar-specific variation, may further strengthen our results and the use of computer vision tools. We, therefore, encourage future studies to directly compare silhouette-based area measurements with traditional weight-estimation techniques, such as volume-based approaches, to quantify both measurement and predictive error in the resulting body-mass models.

## Data Availability

All images, code and statistical analysis of results for the current study are publicly available upon acceptance at https://doi.org/10.5061/dryad.n02v6wxcb.
